# Retrospective analysis of sex-disaggregated immune responses to ALVAC-HIV and bivalent subtype C gp120/MF59 HIV vaccines

**DOI:** 10.3389/fimmu.2025.1557009

**Published:** 2025-05-14

**Authors:** Cassie G. Ackerley, Srilatha Edupuganti, Chenchen Yu, Alison C. Roxby, Kelly E. Seaton, Linda-Gail Bekker, Mary Allen, Stephen C. DeRosa, Nicole L. Yates, Jack Heptinstall, Nonhlanhla N. Mkhize, Mookho Malahleha, Kathryn Mngadi, Brodie Daniels, Craig Innes, Briana D. Furch, Marguerite Koutsoukos, Guido Ferrari, Lynn Morris, David C. Montefiori, M. Juliana McElrath, Georgia D. Tomaras, Fatima Laher, Zoe Moodie

**Affiliations:** ^1^ Department of Medicine, Division of Infectious Diseases, Emory University School of Medicine, Atlanta, GA, United States; ^2^ Vaccine and Infectious Disease Division, Fred Hutchinson Cancer Center, Seattle, WA, United States; ^3^ Duke Human Vaccine Institute and Department of Surgery, Duke University Medical Center, Durham, NC, United States; ^4^ Desmond Tutu HIV Centre, University of Cape Town, Cape Town, South Africa; ^5^ Vaccine Research Program, Division of AIDS, National Institute of Allergy and Infectious Diseases, National Institutes of Health, Bethesda, MD, United States; ^6^ Setshaba Research Centre, Soshanguve, South Africa; ^7^ Aurum Institute, Tembisa Clinic 4, Ekurheleni, South Africa; ^8^ South African Medical Research Council, Durban, South Africa; ^9^ Aurum Institute, Klerksdorp Research Centre, Klerksdorp, South Africa; ^10^ GlaxoSmithKline (GSK) Vaccines, Rixensart, Belgium; ^11^ National Institute for Communicable Diseases, National Health Laboratory Service, Johannesburg, South Africa; ^12^ Perinatal HIV Research Unit, Faculty of Health Sciences, University of Witwatersrand, Johannesburg, South Africa

**Keywords:** sex, immunogenicity, vaccine, HIV, South Africa

## Abstract

**Introduction:**

Generally, individuals assigned female at birth (AFAB) develop greater immunogenicity to various vaccines than individuals assigned male at birth (AMAB). Little is known about sex-disaggregated immunogenicity to HIV-1 vaccines. We disaggregated immune responses to an experimental HIV vaccine regimen.

**Methods:**

We retrospectively analyzed data from HVTN 100, a clinical trial conducted in South Africa during which 143 adults AMAB and 109 AFAB aged 18–40 years without HIV received ALVAC-HIV vCP2438 plus bivalent subtype C gp120/MF59 or placebo at 0, 1, 3, 6, and 12 months. Eligible data were from per-protocol vaccine recipients at month 6.5. We measured IgG binding antibodies, neutralizing antibodies, antibody-dependent cell-mediated cytotoxicity (ADCC), antibody-dependent cellular phagocytosis (ADCP), and CD4+ IFNγ and/or II-2 responses. We compared sex-based differences in response rates using Barnard’s test and response magnitudes using Wilcoxon Rank Sum test. P-values were Holm-adjusted for multiple comparisons.

**Results:**

Of 185 vaccine recipients, 73 were AFAB and 112 were AMAB. Vaccine recipients AFAB had greater ADCC response rate (57.5% versus 29.5%; *p_adj_* = 0.0003) and greater ADCC magnitude (area under the net % granzyme B activity vs log10 curve (AUC), 16.1 versus 11.2; *p_adj_* = 0.05) to vaccine-matched antigen TV1.C gp120 compared to AMAB. Vaccine recipients AMAB had higher CD4+ T cell response rates to 2/3 vaccine-matched antigens at month 6.5 (ZM96.C gp120, [54.1% versus 36.8%; *p_adj_* = 0.04]; 1086.C gp120, [44.1% versus 29.4%; *p_adj_* = 0.05]) than AFAB. CD4+ T cell response magnitudes were similar by sex. IgG binding antibody response rate to B.CaseA V1V2 antigen (associated with reduced HIV acquisition risk in the RV144 trial) was 56.8% among AMAB vaccine recipients versus 38.9% among AFAB (*p_adj_* = 0.08). There were no sex-based differences in neutralizing antibody or ADCP responses.

**Discussion:**

We identified sex-based differences in immune responses to an HIV vaccine regimen, but they varied by immunologic assay. While vaccine recipients AFAB demonstrated higher ADCC responses, AMAB exhibited higher CD4+ T cell response rates. Future analyses should investigate whether vaccine factors such as platform, dosing and adjuvants contribute to sex-based differences in immunogenicity of experimental HIV vaccines.

## Introduction

Vaccine-induced immunogenicity, which may be used to inform vaccine design and as a proxy estimate of efficacy, may differ by sex assigned at birth (1). For many vaccines, individuals assigned female at birth (AFAB) develop higher antibody responses than those assigned male at birth (AMAB), including higher virus-specific antibody responses to measles, mumps, rubella, influenza, hepatitis A and B, herpes simplex virus type 2, rabies, smallpox, and dengue viruses after vaccination (1–3).

Sex difference in immune responses is attributed to multiple factors, including the influence of genes, hormones, and the microbiome on immune regulation (4). Sex is determined by X and Y chromosome composition. The X chromosome plays a crucial role in modulating immune responses and encoding proteins that function in the recognition of foreign antigens (*Toll-like receptor [TLR] 7, TLR8*) (5),cytokine receptor production (*IL2RG, IL13RA2*), and cell differentiation (*FOXP3*) (6). In individuals AFAB, one X chromosome is typically silenced yet 15-20% of genes on this chromosome escape inactivation. This results in differential expression of X-linked genes between sexes and contributes to sex differences in immune function (4). Furthermore, although some immunologic sex differences are lifelong, others appear in the reproductive years, which supports the influence of sex hormones on immune response (7). Sex hormones attach to nuclear receptors which influence transcriptional activity of the cell (8, 9).Estrogen has been shown to enhance antibody production (10), while testosterone may suppress certain immune responses (11). Moreover, there are sex differences from birth in the diversity and quantity of different gut microbiome species, known to modulate immune function and vaccine-induced immune responses, including those to influenza and COVID-19 vaccines (3).

Understanding sex-based disparities in immunogenicity may help inform vaccine development, particularly for diseases with sex-disproportionate morbidity and mortality (4). Sex differences in HIV acquisition are well documented (12). While both sexes are affected, more individuals AFAB are affected – particularly in sub-Saharan Africa (13). Therefore, understanding sex differences in immune responses is relevant to HIV vaccine development.

In this study, we disaggregated immunogenicity by sex to the subtype C ALVAC-HIV and bivalent subtype C gp120/MF59 HIV-1 vaccine regimen among vaccine recipients at the primary immunogenicity timepoint, 2 weeks after the fourth vaccination (month 6.5). Additionally, we determined if there were sex-based differences in vaccine and placebo recipients in T cell responses to cytomegalovirus (CMV) phosphoprotein (pp65) and to Staphylococcal Enterotoxin B (SEB), commonly used as positive control antigens in T cell intracellular cytokine staining (ICS) assays.

## Materials and methods

### Summary of HVTN 100 clinical trial

**Figure 1:**
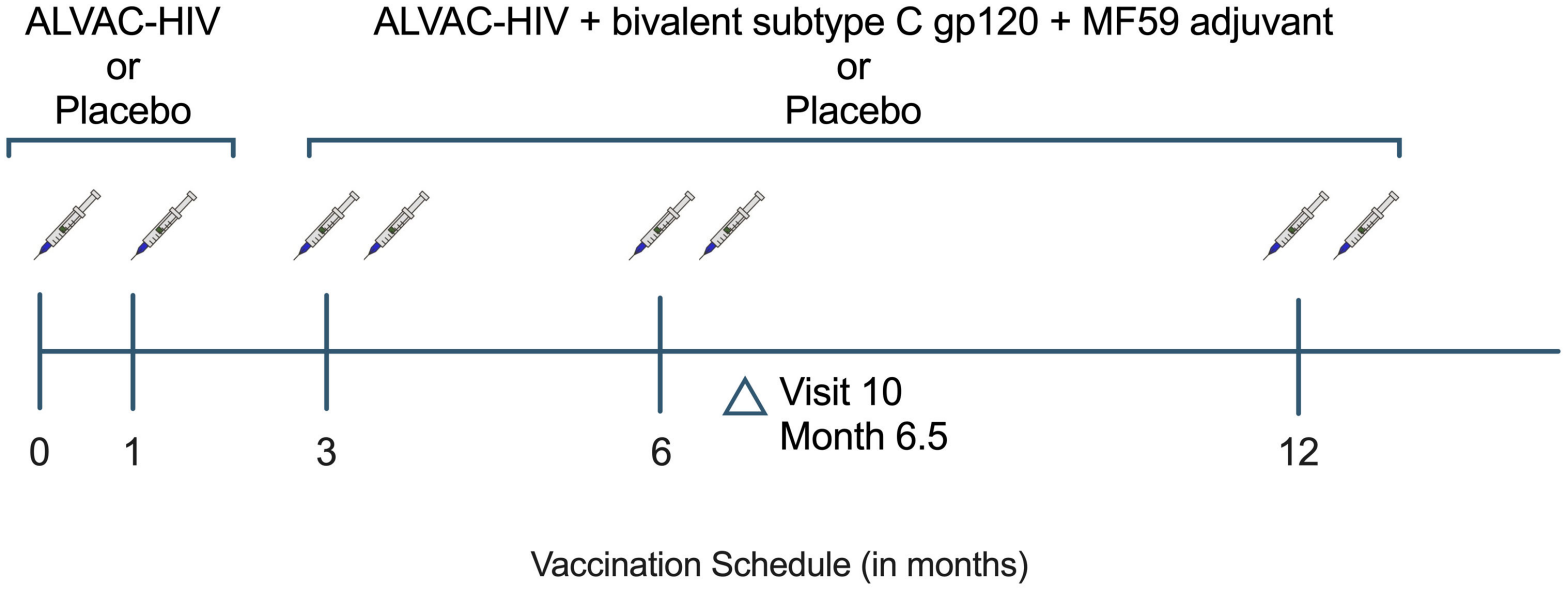
HVTN 100 study schema. Per-protocol participants are defined as having received the first 4 scheduled vaccinations. HVTN, HIV Vaccine Trials Network.

The HVTN 100 trial was a phase 1–2 randomized, placebo-controlled, double-blind trial conducted at 6 community sites in South Africa. Healthy participants aged 18–40 years, not living with HIV, and deemed low vulnerability to HIV acquisition, were enrolled between February and May 2015 and were randomized to receive vaccine or placebo in a 5:1 ratio as previously described (14). Placebo recipients received injections at months 0, 1, 3, 6, and 12 (**Figure 1**). Vaccinees received ALVAC-HIV (vCP2438) at months 0 and 1 followed by ALVAC-HIV (vCP2438) plus bivalent subtype C envelope gp120 and MF59 adjuvant at months 3, 6 and 12.

### Participant data

Data which were eligible for analysis included all available immunogenicity data that met assay-specific quality control criteria. We evaluated immunogenicity data from per-protocol AMAB and AFAB participants from the month 6.5 timepoint, i.e. 2 weeks after the fourth dose of the vaccine regimen. The primary variables included Env-binding IgG and IgG3 antibodies, HIV-1 neutralizing antibodies, antibody-dependent cellular cytotoxicity (ADCC), antibody-dependent cellular phagocytosis (ADCP), and Env-specific CD4+ T cell responses.

### Immunogenicity assays

#### HIV-1 Env-specific IgG and IgG3 binding antibody responses

HVTN 100 serum HIV-1-specific immunoglobulin G (IgG) binding antibody responses were measured in a Good Clinical Laboratory Practice (GCLP) Laboratory using a custom HIV-1 antibody multiplex assay on a Bio-Plex instrument (Bio-Rad) with positive control tracking using Levey-Jennings charts (15). The samples from month 6.5 were measured at dilutions of 1:100 (IgG to V1V2 antigens), 1:200 (IgG to gp120 antigens) or 1:40 (IgG3 to all antigens). Assay readout was background (assay and sample-specific) subtracted mean fluorescence intensity (MFI). Negative controls included HIV-1 seronegative human serum. Samples were excluded from the analysis if the blood draw occurred outside of the allowable visit window or if the blank bead negative control exceeded 5,000 MFI on two subsequent assay runs. Positive responses were determined if three conditions were met: (1) the MFI minus Blank values were ≥ antigen-specific cutoff at the 1:50 dilution level (based on the 95^th^ percentile of the baseline visit serum samples and at least 100 MFI), (2) the MFI minus Blank values were greater than 3 times the baseline (day 0) MFI minus Blank values, and (3) the MFI values were greater than 3 times the baseline MFI values. IgG and IgG3 binding antibody response magnitudes are summarized as netMFI. Antigens were chosen to represent vaccine-matched sequences, to represent antigens identified as correlates of HIV acquisition risk in the RV144 trial (B.CaseA V1V2), or consensus sequences (Con 6 gp120/B) (16).

Magnitude-breadth IgG and IgG3 scores were performed in this study. This score is a summary measure that describes the magnitude and breadth across a panel of antigens and represents the area under the magnitude-breadth curve for an individual vaccine recipient, as previously described (17).

#### Neutralizing antibody responses

Neutralization titers at 50% inhibitory dilution (ID50) were measured as a function of reductions in Tat-regulated luciferase (Luc) reporter gene expression in TZM-bl cells. For this analysis, neutralization titers against two heterologous Env-pseudotyped Tier 1a viruses were assessed, MW965.26 (non-vaccine-matched) and TV1c8.2 (vaccine-matched). For the assay, a titer is defined as the serum dilution that reduces relative luminescence units (RLUs) by 50% relative to the RLUs in virus control wells (cells incubated with virus) after subtraction of the background RLU (cells only). In this neutralization assay utilizing TZM-bl cells, response to a virus was considered positive if the neutralization titer was above the pre-specified cutoff of 10. Response magnitude plots characterize the neutralization titer (ID50) of each individual serum sample and results are stratified based on sex assigned at birth.

#### Antibody-dependent cellular cytotoxicity responses

Participant sera were incubated with effector cells and vaccine-matched gp120 coated target cells (ZM96 gp120, 1086.C gp120, and TV1.C gp120). ADCC-mediated antibody responses were quantified as net percent granzyme B activity (18). Net percent granzyme B activity was measured by the difference between the percent of target cells positive for GranToxiLux (GTL), an indicator of granzyme B uptake, and the percent of target cells positive for GTL when incubated with effector cells and no sera. Flow cytometry was used to quantify the frequency of granzyme B positive cells in the assay. For each participant, percent granzyme B activity was measured at 6 dilution levels (50, 250, 1250, 6250, 31,250 and 156,250) for each antigen. ADCC magnitude was measured as area under the net percent granzyme B activity versus log10 dilution curve (AUC) and calculated using the trapezoidal rule. A positive response was defined as peak granzyme B activity greater than or equal to 10%.

#### Antibody-dependent cellular phagocytosis responses

Neutravidin fluorescent beads coated with 1086gp140 were first incubated with diluted sera and control monoclonal antibodies (positive control CH58 and negative control CH65). THP-1 cells and human monocytic effector cells (pre-treated with anti-human CD4 to reduce CD4-Env mediated virus internalization) were incubated with the antibody/HIV-1 protein conjugated microsphere complexes, and then paraformaldehyde-fixed prior to analysis by flow cytometry. The phagocytic scores were determined based on the ratio of experimental sample to a control sample with no antibody. Positive responses were determined if both of the following criteria were met: (1) Mean phagocytosis score at follow-up ≥ positivity threshold and (2) Mean phagocytosis score at follow-up ≥ 3 x mean phagocytosis score at baseline.

#### Intracellular cytokine staining

The homologous peptide pools evaluated and included in this study were comprised of 15-mer peptides overlapping by 11 amino acids across 1086 gp120, TV1 gp120, and ZM96 gp120. The magnitude of CD4+ and CD8+ T cell responses were measured by intracellular cytokine staining (ICS) assessing expression levels of IFN-γ and/or IL-2. Cryopreserved peripheral blood mononuclear cells (PBMCs) determined to have a viability of ≥66% were stimulated *ex vivo* with vaccine-matched synthetic pooled peptides. For these analyses, the mean PBMC viability was 92% with a standard deviation of 3.83. Unstimulated cells were utilized as negative controls. For positive controls, cells were stimulated with cytomegalovirus pp65 (CMV) and staphylococcal enterotoxin B (SEB). Samples with fewer than 5000 CD4+ or CD8+ T cells were excluded from the ICS analysis. A positive response of a peptide pool within a T cell subset was determined by one-sided Fisher’s exact test applied to the peptide pool response versus the negative control response with discrete Bonferroni-Holm adjustment for multiple comparisons. If the adjusted p-value ≤ 0.00001, the response to the peptide pool for the corresponding T-cell subset was considered positive. The background-adjusted percentage of CD4+ and CD8+ T cells expressing IFN-γ and/or IL-2 was analyzed, where this net percent was calculated as % of antigen-stimulated cells minus % of unstimulated, negative control cells.

Functionality and polyfunctionality scores for antigen-specific T cell subsets were analyzed by COMPASS (Combinatorial Polyfunctionality Analysis of Antigen-Specific T-cell Subsets). The functionality score is determined by the estimated proportion of subsets most likely to have antigen-specific responses among all possible subsets, and the polyfunctionality score is similar but weighs the different subsets by their degree of functionality (19). COMPASS probabilities were reported for all observed CD4+ T cell subsets (interferon-gamma, interleukin-2, tumor necrosis factor alpha, CD40 ligand, interleukin-4, and granzyme B).

### Statistical methods

Immunogenicity data from per-protocol participants were included in our analyses. All immune assays were conducted in HVTN laboratories by staff blinded to participant study product assignment.

Response rates are the proportion of participants with positive immune assay response among the total participants within the sex-based cohort. Response rate data is displayed using bar graphs and the 95% Wilson confidence intervals (CI) are provided in **Supplementary Tables S1** and **S2**. Response magnitudes for each immune assay among AFAB and AMAB participants are displayed using boxplots where the box edges reflect the 25^th^ and 75^th^ percentiles or interquartile range (IQR), the midline denotes the median, and the whiskers extend to the minimum and maximum data points. Response rates between groups (AFAB versus AMAB) were compared using Barnard’s exact test. Response magnitudes were compared between groups using the Wilcoxon rank sum test. A *p* value of <0.05 was used to determine statistical significance. Within each immune assay type, *p* values were adjusted for multiple comparisons using Holm-Bonferroni correction. For data plotted using a log10 scale, zero and negative values were converted to a standard minimum value. Statistical analyses were performed using R (Version 4.3.2). Figures were prepared using GraphPad Prism 10.4.1.

## Results

### Trial cohorts

Of the 252 participants enrolled in the HVTN 100 trial, 222 were included in the per-protocol cohort, which consisted of participants who received the first four vaccine doses and did not acquire HIV by month 6.5. In the per-protocol cohort (disaggregated by sex in **Table 1**), 185 (83%) participants received vaccine, 37 (17%) received placebo, while 131 (59%) were AMAB and 91 (41%) were AFAB.

**Table 1:** Characteristics of the per-protocol cohort (n = 222), stratified by sex.

Characteristic	AFAB (n = 91)	AMAB (n = 131)	Total (n = 222)
Study product assignment, n (%)			
Vaccine Placebo	73 (80.2)	112 (85.5)	185 (100%)
	18 (19.8)	19 (14.5)	37 (100%)
Age, median (IQR)	23 (20-27)	23 (21-26)	

AFAB, Assigned female at birth; AMAB, Assigned male at birth; IQR, Inter-quartile range.

### Few sex-based differences in IgG and IgG3 binding antibody response rates or magnitudes among vaccine recipients

**Figure 2:**
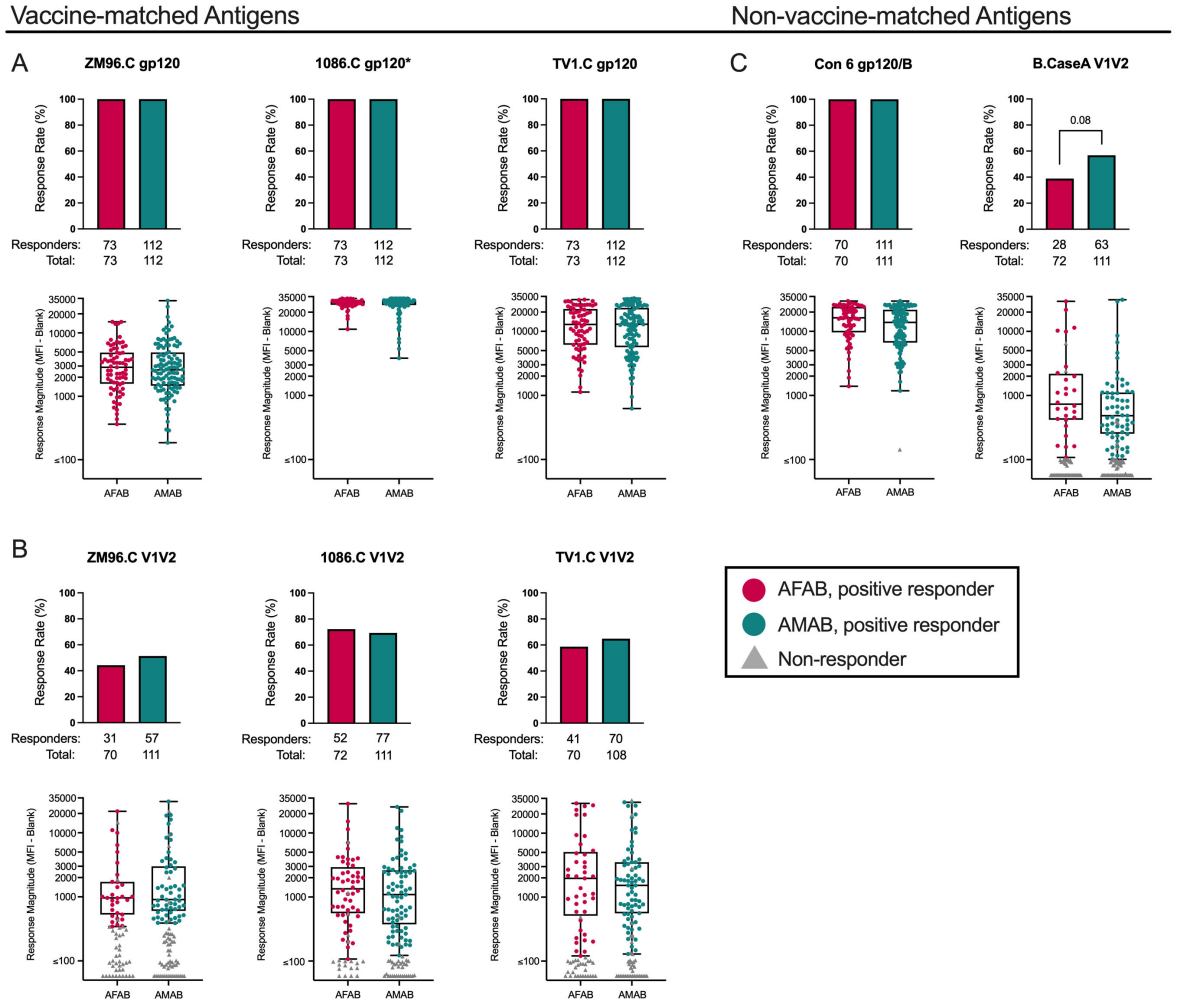
IgG binding Ab response rates and magnitudes among AFAB and AMAB vaccine recipients. No significant sex-based differences in binding IgG response rates or magnitudes to **(A)** vaccine-matched gp120 antigens or **(B)** vaccine-matched V1V2 antigens were observed. Among the **(C)** non-vaccine matched antigens, there was a trend toward a higher response rate to B.CaseA V1V2 antigen, a protein associated with reduced vulnerability to HIV acquisition in the RV144 trial, among AMAB vaccine recipients compared to AFAB. Response rates are shown using bar graphs. Boxplots of positive responders show magnitude as MFI-Blank responses. *P* values compare response rates and magnitudes among positive responders – AFAB positive responders (shown in red circles), AMAB positive responders (shown in dark green circles); negative responders are shown as gray triangles. Adjusted *p*-values reported. The asterisk symbol (*) indicates that statistical comparisons based on sex were not performed as the median netMFIs for AFAB and AMAB vaccine recipients exceeded the upper limit of the linear range of the assay (>22,000). AFAB, assigned female at birth; AMAB, assigned male at birth; gp120, glycoprotein 120; IgG, immunoglobulin G; V1V2, Variable loops 1 and 2.

Using sera from per-protocol vaccine recipients, we measured IgG and IgG3 binding antibody responses to 3 gp120 vaccine-matched antigens (ZM96.C, 1086.C, and TV1.C), 3 V1V2 vaccine-matched antigens (ZM96.C, 1086.C, and TV1.C), and 2 non-vaccine-matched antigens (Con 6 gp120/B and B.CaseA V1V2). We compared vaccine-elicited IgG and IgG3 binding antibody response rates and magnitudes by sex assigned at birth. In our analysis of IgG binding antibody responses, 73 AFAB (100% [95% CI 95.0-100%]) and 112 AMAB (100% [95% CI 96.7-100%]) vaccine recipients developed positive IgG binding antibody responses to all three vaccine-matched subtype C gp120 antigens at month 6.5 (**Figure 2A**, **Supplementary Table S1**). There were no differences by sex in the magnitude of IgG binding antibody responses to ZM96.C or TV1.C gp120 antigens. The median netMFIs for AFAB and AMAB vaccine recipients to 1086.C gp120 exceeded the upper limit of the linear range of the assay (>22,000) at the dilution tested, so statistical comparison by sex was not performed.

Response rates to the 3 vaccine-matched V1V2 antigens were lower than responses to vaccine-matched gp120 antigens (**Figure 2B**, **Supplementary Table 1**). There were no sex-based differences in IgG binding antibody response rates or magnitudes to the vaccine-matched V1V2 antigens. (**Supplementary Table S1**).

We also evaluated vaccine recipients for IgG binding antibody responses to Con 6 gp120/B and B.CaseA V1V2 as non-vaccine-matched antigen comparators (**Figure 2C**, **Supplementary Table S1**). In the RV144 trial, B.CaseA V1V2 had been identified as an immune correlate of protection from HIV-1 acquisition (20). At month 6.5, AMAB participants had an IgG binding antibody response rate of 56.8% (95% CI 47.5-65.6%) versus AFAB 38.9% ([95% CI 28.5-50.4%]; *p* = 0.01, *p_adj_* = 0.08). Despite fewer positive AFAB responders, netMFIs were greater among AFAB compared to AMAB positive responders (731 [IQR 425.6-2047.2] versus 484 [IQR 258.2-1109.8]), but this comparison did not remain statistically significant after adjustment for multiple comparisons (*p* = 0.04, *p_adj_* = 0.26). For Con 6 gp120/B, there was a similar pattern as seen for the vaccine-matched gp120 antigens, specifically a high response rate for AFAB and AMAB vaccine recipients. AFAB vaccine recipients demonstrated higher netMFIs among positive responders compared to AMAB (16293 [IQR 9923.1-24008.4] versus 13792 [IQR 6680.1-21726.1]; *p* = 0.02, *p_adj_* =0.15), but this difference was not significant after multiplicity adjustment.

In our assessment of IgG3 binding antibody responses, there were no sex-specific differences in IgG3 response rates or response magnitudes (**Supplementary Figure S1**, **Supplementary Table S1**). Additionally, we evaluated the magnitude-breadth of IgG and IgG3 binding antibody responses utilizing pre-specified antigen panels (gp120, gp140, and V1V2) to examine global vaccine coverage (21). There were no differences in the breadth of IgG or IgG3 responses based on sex (**Supplementary Table S1**).

### No sex-based differences in neutralizing antibody responses among vaccine recipients

**Figure 3:**
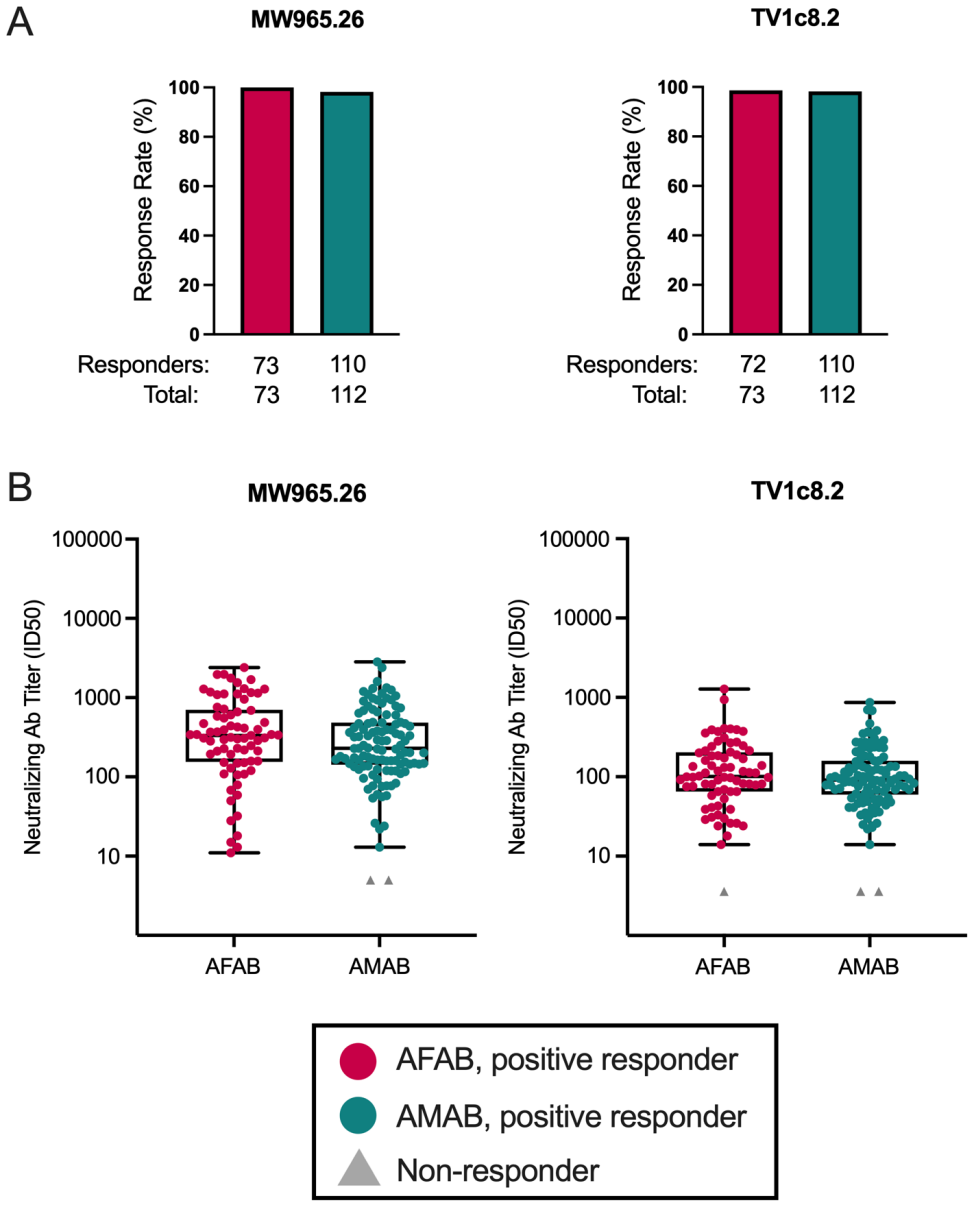
Neutralizing antibody response rates and magnitudes among AFAB and AMAB vaccine recipients. There were no sex-based differences in neutralizing antibody **(A)** response rates or **(B)** response magnitudes to two Tier 1A viruses between AMAB and AFAB vaccine recipients. Response rates are shown using bar graphs. Boxplots of positive responders show magnitude as ID50 responses. *P* values compare response rates and magnitudes among positive responders – AFAB positive responders (shown in red circles), AMAB positive responders (shown in dark green circles); negative responders are shown as gray triangles. Adjusted *p*-values < 0.05 are reported. Ab, antibody; AFAB, assigned female at birth; AMAB, assigned male at birth; ID50 – 50% inhibitory dilution.

We evaluated sex-based differences in response rates and neutralization titers (ID50) against two heterologous Env-pseudotyped Tier 1a viruses, one non-vaccine-matched (MW965.26) and the other a vaccine-matched clade C strain (TV1c8.2). Response rates greater than 98% to MW965.26 and TV1c8.2 were observed among AFAB and AMAB participants (**Figure 3A**; **Supplementary Table S1**). We did not find any differences in neutralization titers among positive responders by sex assigned at birth for the two Tier 1a viruses (**Figure 3B**, **Supplementary Table S1**).

### Higher ADCC response rate and magnitude to TV1.C gp120 among AFAB vaccine recipients

**Figure 4:**
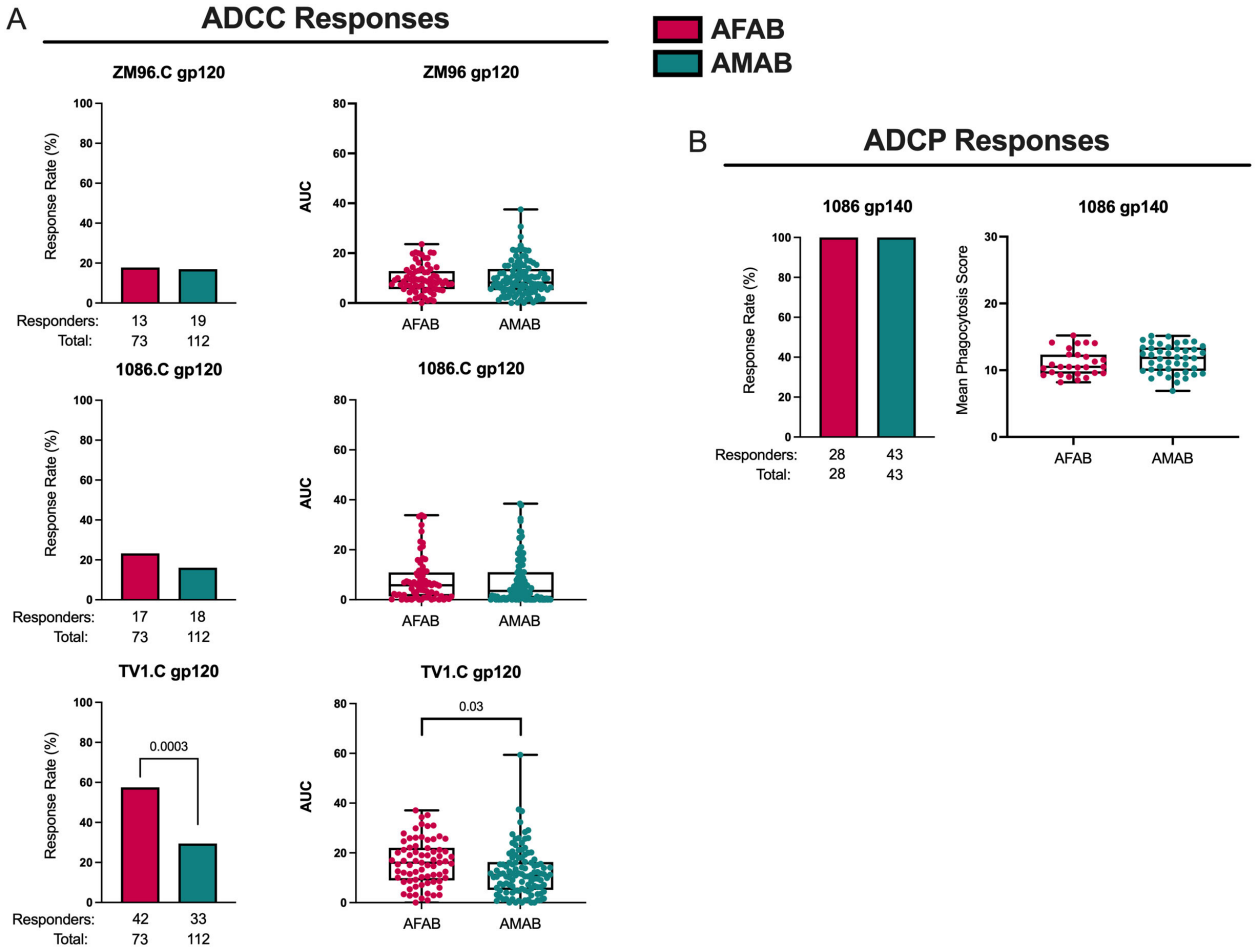
ADCC and ADCP response rates and magnitudes among AFAB and AMAB vaccine recipients. **(A)** AFAB vaccine recipients had higher ADCC response rate and AUC to TV1.C gp120. **(B)** No sex-based differences in vaccine-induced ADCP response rates or magnitudes were observed. Response rates are shown using bar graphs. Boxplots of positive responders are used to demonstrate response magnitudes. *P* values compare response rates and magnitudes among positive responders – AFAB positive responders (shown in red circles), AMAB positive responders (shown in dark green circles); negative responders are shown as gray triangles. Adjusted *p*-values <0.05 are reported. AFAB, assigned female at birth; AMAB, assigned male at birth; AUC, area under the curve; gp, glycoprotein.

AFAB vaccine recipients (57.5% [95% CI 46.1-68.2%], n = 42 responders) demonstrated a greater ADCC response rate, based on peak granzyme B activity, to TV1.C gp120 compared to AMAB (29.5% [95% CI 21.8-38.5%], n = 33 responders) at month 6.5 (*p* = 8.44e-05, *p_adj_* = 0.0003; **Figure 4A**, **Supplementary Table S1**). There was also a higher ADCC AUC response magnitude among AFAB vaccine recipients to TV1.C (16.1 [IQR 9.2-21.9] versus 11.2 [IQR 5.4-16.2]; *p* = 0.009, *p_adj_* = 0.03) and a trend toward a greater ADCC AUC response magnitude to 1086.C gp120 (5.8 [IQR 1.4-10.5] versus 3.5 [IQR 0.7-10.9]; *p* = 0.05, *p_adj_* = 0.10) compared to AMAB vaccine recipients. All AFAB and AMAB vaccine recipients showed positive ADCP responses (**Figure 4B**, **Supplementary Table S1**), with no sex-based differences in the magnitude of average phagocytosis scores.

### Trend toward higher CD4+ T cell response rates among AMAB vaccine recipients, but similar response magnitudes among positive responders by sex

**Figure 5:**
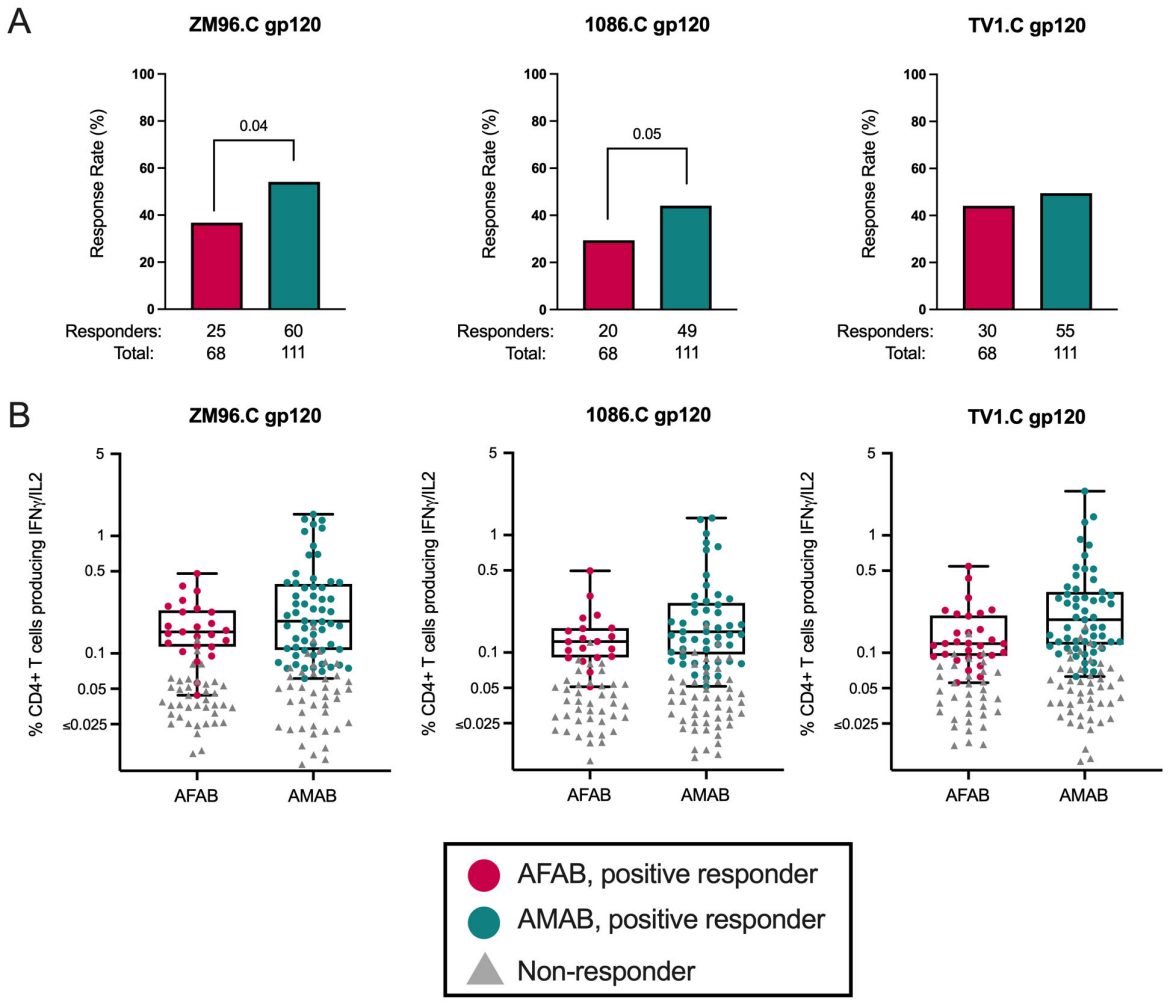
CD4+ T cell response rates and magnitudes among AFAB and AMAB vaccine recipients. **(A)** AMAB vaccine recipients had higher response rates to ZM96 and 1086C gp120 antigens compared to AFAB vaccine recipients. **(B)** There were no significant differences in CD4+ T cell response magnitudes based on sex assigned at birth. Response rates are shown using bar graphs. Boxplots show magnitude as % CD4+ T cells producing IFN-γ and/or IL-2. *P* values compare response rates and magnitudes among positive responders – AFAB positive responders (shown in red circles), AMAB positive responders (shown in dark green circles); negative responders are shown as gray triangles. Adjusted *p*-values reported. AFAB, assigned female at birth; AMAB, assigned male at birth; gp120, glycoprotein 120; IFN-γ, interferon gamma; IL-2, interleukin-2.

Next, we compared vaccine-induced CD4+ T cell ICS responses among AFAB and AMAB vaccine recipients. We found that AMAB vaccine recipients had a higher response rate to ZM96.C gp120 (54.1% [95% CI 44.8-63.0%] versus 36.8% [95% CI 26.3-48.6%]; *p* = 0.01, *p_adj_* = 0.04) and a trend toward a greater response rate to 1086.C gp120 (44.1% [95% CI 35.3-53.4%] versus 29.4% [95% CI 19.9-41.1%]; *p* = 0.03, *p_adj_* = 0.05) compared to AFAB vaccine recipients (**Figure 5A**, **Supplementary Table S1**). There were no significant sex-based differences in CD4+ ICS response magnitudes among positive responders to the 3 vaccine-matched antigens (**Figure 5B**, **Supplementary Table S1**). Because CD8+ T cell response rates were <5% among vaccine recipients, these responses were not analyzed. In a comparison of functionality and polyfunctionality scores for CD4+ T cell responses, there were no sex-specific differences in scores for any vaccine-matched antigens (**Supplementary Table S1**).

### Similar CD4+ and CD8+ T cell ICS responses to CMV and SEB among AFAB and AMAB vaccine and placebo recipients

**Figure 6:**
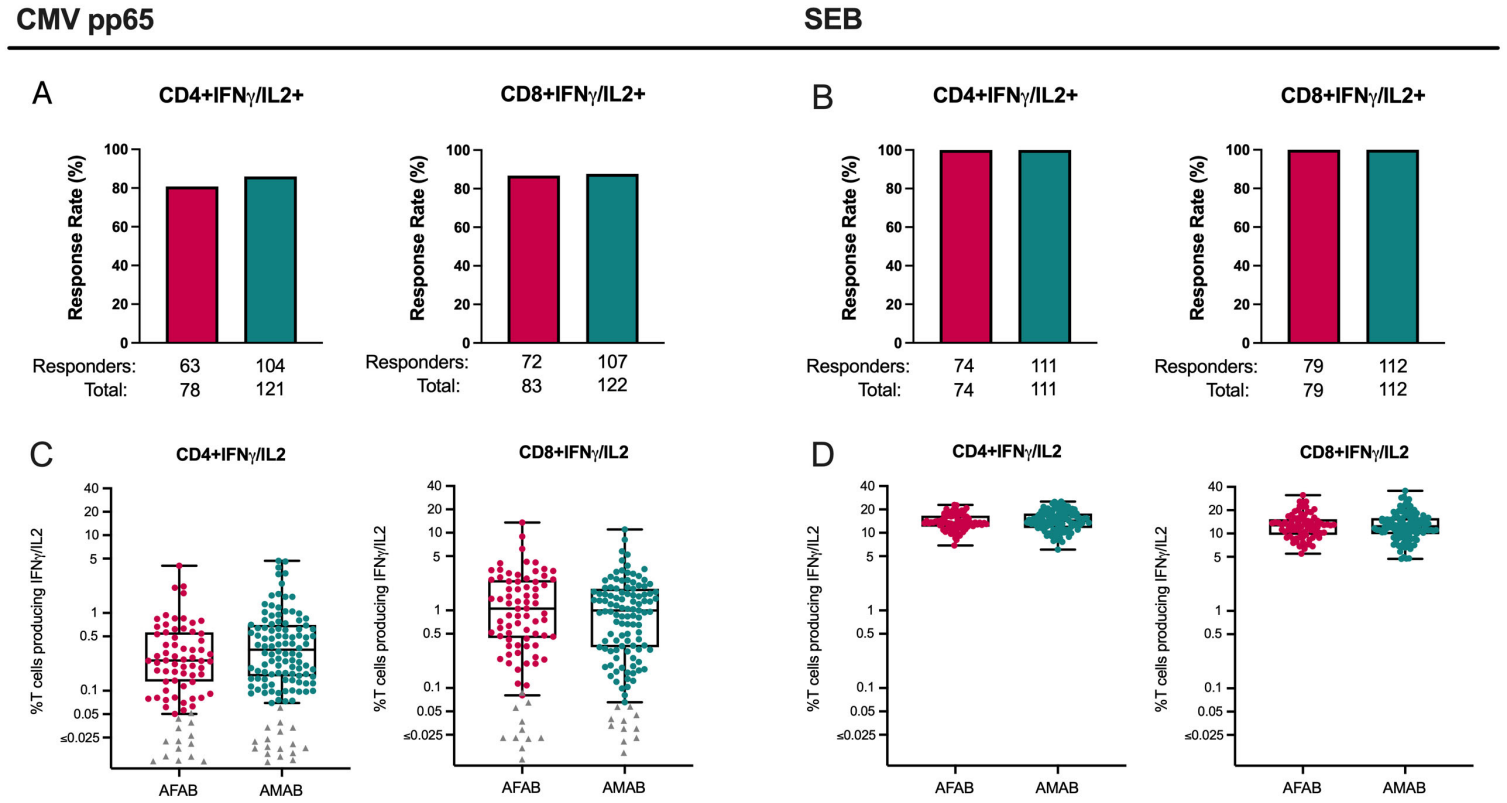
CD4+ and CD8+ T cell responses among AMAB and AFAB vaccine and placebo recipients to CMV and SEB. There were no significant sex-based differences in CD4+ or CD8+ response rates **(A, B)** or magnitudes **(C, D)** to CMV or SEB control antigens. Response rates are shown using bar graphs. Boxplots of positive responders show magnitude as CD4+ or CD8+ T cells producing IFN-γ and/or IL-2. *P* values compare response rates and magnitudes among positive responders – AFAB positive responders (shown in red circles), AMAB positive responders (shown in dark green circles); negative responders are shown as gray triangles. Adjusted *p*-values < 0.05 are reported. AFAB, assigned female at birth; AMAB, assigned male at birth; CMV, cytomegalovirus; gp120, glycoprotein 120; IFN-γ, interferon gamma; IL-2, interleukin-2; SEB, staphylococcal endotoxin B.

CD4+ and CD8+ T cell response data to ICS assay control antigens (i.e., CMV pp65 and SEB) from both per-protocol vaccine and placebo recipients were assessed for baseline differences in T cell responses based on sex assigned at birth and unrelated to vaccine exposure. There were no meaningful sex-based differences in CD4+ or CD8+ T cell response rates or magnitudes to CMV pp65 or SEB (**Figures 6A–D**, **Supplementary Table S2**).

## Discussion

We found some sex-based differences in vaccine-induced immunogenicity to the subtype C ALVAC-HIV and bivalent subtype C gp120/MF59 experimental HIV-1 vaccine regimen, but they varied by immunologic assay. AFAB vaccine recipients had a greater ADCC response rate and magnitude to one of the vaccine-matched antigens compared to AMAB. In contrast, AMAB vaccine recipients demonstrated higher CD4+ T cell response rates to 2 of the 3 vaccine-matched antigens, although response magnitudes were similar among AFAB and AMAB positive responders. While there were minimal sex-based differences in IgG and IgG3 binding Ab response rates or magnitudes, AMAB did have a higher proportion of positive responders to B.CaseA V1V2 antigen, previously associated with reduced vulnerability to HIV acquisition in the RV144 clinical trial (20). Additionally, we found no sex-based differences in neutralizing antibody or ADCP responses and no differences in T cell responses among AMAB and AFAB vaccine and placebo recipients to CMV pp65 or SEB, positive control antigens used in T cell ICS assays.

ADCC plays an important role in antiviral immunity, occurring when antibody forms a bridge between a target viral-infected cell and a Fc receptor-bearing effector cell resulting in lysis of the target cell (22). In some non-human primate and human SIV/HIV vaccine studies, antibody-dependent, cell-mediated virus inhibition antibody responses were correlated with protection from SIV/HIV acquisition (23–25).Despite evidence supporting the importance of ADCC responses in antiviral immunity, there is little data examining how sex-associated biologic factors, including sex chromosomal composition and sex hormone milieu, may influence vaccine-induced immunity. In a pediatric study conducted in The Gambia, AMAB infants were found to have greater measles virus-specific ADCC responses following vaccination with Edmonston-Zagreb measles vaccine compared to AFAB infants, although this difference was observed only among infants with no pre-immunization anti-measles antibodies (26). Notably, sex-based differences in ADCC responses after immunization were not seen among infants who received a different type of measles vaccine (Schwarz vaccine), suggesting that vaccine platforms can have variable effects on the development of vaccine-induced ADCC immunity. In a study evaluating ADCC activity after SARS-CoV-2 infection and vaccination, differences in ADCC responses were observed based on sex assigned at birth. Specifically, AMAB participants showed higher ADCC responses against S and N proteins compared to AFAB when all assessed samples were pooled (27). In our study, we found a higher ADCC response rate and magnitude to TV1.C gp120 among AFAB compared to AMAB vaccine recipients. As fewer than 60% of vaccine recipients in each sex-based cohort had positive ADCC assay responses in this study, the sample size of positive responders may have limited our ability to detect further differences between groups. Additional data is needed to better characterize sex-specific antibody-dependent cell-mediated immune responses and to evaluate the impact of different types of vaccine platforms on this aspect of vaccine-induced immunity.

In this study, we observed that AMAB vaccine recipients demonstrated a higher CD4+ T cell response rate to ZM96.C gp120 and a trend toward a greater response to 1086.C gp120 compared to AFAB. Also, in our analysis of IgG antibody responses, AMAB vaccine recipients had a trend toward a greater response rate to B.Case A V1V2 compared to AFAB vaccine recipients. Yet, the frequencies of CD4+ T cells expressing IFN-γ and/or IL-2 in response to the 3 vaccine-matched antigens and the IgG/IgG3 response magnitudes did not differ based on sex. Prior immunization studies have predominantly shown greater vaccine-induced immune responses among AFAB vaccine recipients, yet there are studies where humoral and cell-mediated immune responses have prevailed among AMAB vaccinees (28). In an assessment of sex-based differences in immune responses at the week 26 timepoint for RV144 study participants, male vaccine recipients were found to have increased frequencies of CD16-CD56dim natural killer cells, myeloid dendritic cells, and plasmacytoid dendritic cells compared to females (29). As we did not evaluate innate immune cell subset frequencies in our study, we were unable to directly compare our results with those from the RV144 trial. Consistent with our findings, there were minimal differences in humoral immune responses to the RV144 ALVAC-HIV (vCP1521) prime and AIDSVAX B/E boost vaccine regimen based on sex assigned at birth (29). In a participant-level meta-analysis of IMVAMUNE clinical trials assessing responses to a live attenuated smallpox vaccine, males were found to generate nearly a 25% higher peak geometric mean antibody titer at 14 days post-vaccination compared to females (30). Another study evaluating smallpox vaccine-induced cellular immune responses between men and women demonstrated significantly higher IFN-γ ELISPOT responses and greater secretion of the pro-inflammatory cytokine IL-1β from stimulated PBMCs among male vaccine recipients compared to females (31). Among a cohort of adolescents who received two doses of MMR-II vaccine, female vaccine recipients were noted to have higher neutralizing antibody titers compared to males, yet the mumps-virus stimulated PBMCs from male participants secreted higher levels of multiple cytokines and chemokines (MIP-1α, MIP-1β, TNF-α, IL-6, IFN-γ, and IL-1β) compared to PBMCs obtained from females (32). As sex-based vaccine-induced immune responses are rarely an outcome of interest in vaccine clinical trials, there remains limited data on how various viral vaccines induce immune responses among AFAB and AMAB participants. This paucity of data makes it exceedingly difficult to establish clinically relevant sex-based differences in vaccine-induced immunity and inhibits our ability to further analyze the underlying mechanisms that may be contributing to these differences, including sex hormone concentrations (i.e., β-estradiol, progesterone, or testosterone) or possibly the effects of sex chromosome composition.

In this study, we found variable sex-based differences in vaccine-induced immune responses to the HVTN 100 vaccine regimen depending on the immunologic assay. Additionally, we found there were no differences based on sex in T cell responses to CMV or SEB, highly immunogenic antigens used as positive controls in the ICS assays, among vaccine and placebo recipients. These findings suggest that utilization of repeat prime vaccine doses, vaccine boosts, and/or robust adjuvants, as well as the immunogenic potential of an antigen, may be able to overcome variability in some sex-specific immune response phenotypes. Yet, it remains highly useful to consider how vaccine components and dosing regimens may alter immune responses based on sex. In a review of 97 studies evaluating 14 different vaccine products, sex-specific differences in vaccine-induced humoral immunity were observed among a variety of vaccine types (i.e., live vs inactivated vaccines) and platforms, including viral vector, protein-based, toxoid vaccines, etc. (33) While the drivers of differential humoral responses to these various vaccines remained unclear, there was a strong recommendation to evaluate for sex-based differences in vaccine clinical trials given the potential clinical implications. Along with vaccine type, sex-specific humoral responses may impact dosing strategy as well. For example, in a study comparing hemagglutination inhibition (HAI) titers following receipt of half- vs full-dose intramuscular trivalent inactivated influenza vaccine, geometric mean antibody titers in females to half-dose were comparable to male full-dose vaccine responses (34). Females were also found to have higher geometric mean antibody titers following receipt of the quadrivalent human papilloma virus (HPV) vaccine (35). Recent data suggests that a single dose of nonavalent or bivalent HPV vaccine among females was highly effective in preventing incident persistent oncogenic HPV infection (36), yet it is less clear whether this dosing strategy would be effective in males due to differential humoral responses to the vaccine. Therefore, sex-specific effects could impact the dosing strategy for a vaccine product and influence economic considerations like the cost-effectiveness of providing half vs full dose or fewer doses of a vaccine regimen based on sex.

Adjuvants are another vaccine component that play an important role in influencing the magnitude and persistence of the humoral response following vaccination (37), yet little is known about how adjuvant performance may vary based on sex assigned at birth. In a mouse model exploring the effect of adjuvant use on sexual dimorphic antibody responses to an inactivated foot-and-mouth disease virus vaccine, use of an oil-in-water adjuvant enhanced IgM antibody responses in female mice but the same effect was not observed among male mice (38). Toll-like receptors (TLRs) have garnered interest as potential vaccine adjuvants that could enhance vaccine efficacy given their role in regulating innate immune responses that further stimulate adaptive immunity (39), and TLR-7 and TLR-8 agonists have been developed for this purpose. Notably, the TLR-7 and TLR-8 genes are encoded on the X chromosome, and 15-20% of human X chromosomal genes have been shown to escape inactivation in AFAB individuals (40). X chromosome inactivation escape results in increased expression of TLR-7 in various immune cells, including B cells, which has been associated with higher antibody production among female mice in response to influenza A vaccination and infection using a murine model (5). TLR-7 agonists have also been shown to induce greater IFN-α secretion among females compared to males (41). Combinations of TLR agonists have been studied in the context of heightening antibody responses to HIV-1 envelope protein, and the combination of TLR7/8 and TLR9 agonists utilized as a vaccine adjuvant stimulated higher levels of ADCC and tier 1 neutralizing antibodies compared to other combinations among non-human primates, although possible differential responses based on sex to this adjuvant combination were not explored (42). Further evaluation is warranted to better understand how sex-specific immunity may impact responses to vaccine regimens depending on the vaccine platform, the dosing schedule, and based on use of certain vaccine adjuvants. With this knowledge, it may be possible to tailor the design of vaccine products to individuals AMAB or AFAB to enhance vaccine efficacy by capitalizing on known sex-specific immune response mechanisms (43).

As this study is a secondary analysis of data from the HVTN 100 clinical trial and sex-based differences were not the primary outcome of interest, AFAB and AMAB sample sizes for some immune assays may be inadequate to reliably determine whether there are sex-based differences in vaccine-induced immune responses. Furthermore, lower response rates to the vaccine regimen, as seen for some immunologic assays (i.e., ADCC and CD8+ T cell responses), limited sex disaggregation. Additionally, in this study, we focus on differences in vaccine-induced immunity based on sex, but we are unable to draw conclusions about whether these variable immune responses could impact vaccine efficacy for a candidate HIV-1 vaccine regimen.

Overall, we found that there were some sex-based differences in immune responses to the subtype C ALVAC-HIV vaccine regimen. Our results support the rationale to disaggregate vaccine-induced immunogenicity by sex. Designing future HIV vaccine trials with a focus on sex-based immune responses as an outcome of interest not only provides insight regarding immune responses to a particular vaccine regimen, but it also greatly contributes to our overall understanding of the underlying mechanisms that drive sex-based immune responses. Considering that HVTN trials are being conducted in multiple countries and among sexual and gender diverse populations, evaluating sex- and gender-based differences in safety, immunogenicity, and efficacy to HIV vaccine regimens will be critical to ensure that those most vulnerable to HIV acquisition will receive the intended benefit.

## Data Availability

The original contributions presented in the study are included in the article/**Supplementary Material**. Further inquiries can be directed to the corresponding author.

## Supplementary material

Supplementary Figure 1 IgG3 binding Ab response rates and magnitudes among AFAB and AMAB vaccine recipients. **(A–C)** There were no sex-based differences in response rates or response magnitudes. Response rates are shown using bar graphs. Boxplots of positive responders show magnitude as MFI-Blank responses. *P* values compare response rates and magnitudes among positive responders – AFAB positive responders (shown in red circles), AMAB positive responders (shown in dark green circles); negative responders are shown as gray triangles. Adjusted *p*-values < 0.05 are reported. AFAB, assigned female at birth; AMAB, assigned male at birth; gp120, glycoprotein 120; IgG, immunoglobulin G; V1V2, Variable loops 1 and 2.

## References

[1] Klein SL Marriott I Fish EN. Sex-based differences in immune function and responses to vaccination. Trans R Soc Trop Med Hyg. (2015) 109:9–15. doi: 10.1093/trstmh/tru167 25573105 PMC4447843

[2] Engler RJM Nelson MR Klote MM VanRaden MJ Huang C Cox NJ. Half- vs full-dose trivalent inactivated influenza vaccine (2004-2005). Arch Intern Med. (2008) 168:2405–15. doi: 10.1001/archinternmed.2008.513 19064822

[3] Rio P Caldarelli M Chiantore M Ocarino F Candelli M Gasbarrini A. Immune cells, gut microbiota, and vaccines: A gender perspective. Cells. (2024) 13:1–26. doi: 10.3390/cells13060526 PMC10969451 38534370

[4] Klein SL Flanagan KL. Sex differences in immune responses. Nat Rev Immunol. (2016) 16:626–38. doi: 10.1038/nri.2016.90 27546235

[5] Fink AL Engle K Ursin RL Tang WY Klein SL. Biological sex affects vaccine efficacy and protection against influenza in mice. Proc Natl Acad Sci U S A. (2018) 115:12477–82. doi: 10.1073/pnas.1805268115 PMC6298067 30455317

[6] Bianchi I Lleo A Gershwin ME Invernizzi P. The X chromosome and immune associated genes. J Autoimmun. (2012) 38:J187–92. doi: 10.1016/j.jaut.2011.11.012 22178198

[7] Sciarra F Campolo F Franceschini E Carlomagno F Venneri MA. Gender-specific impact of sex hormones on the immune system. Int J Mol Sci. (2023) 24. doi: 10.3390/ijms24076302 PMC10094624 37047274

[8] Hammes SR Mendelson CR. Mechanisms of hormone action. In: Textbook of Endocrine Physiology, 6th Edition. Oxford University Press, Inc, New York (2011). p. 58–97. Available at: https://ebookcentral.proquest.com/lib/emory/detail.action?docID=845972 (Accessed October 3, 2024).

[9] Kato S Sato T Watanabe T Takemasa S Masuhiro Y Ohtake F. Function of nuclear sex hormone receptors in gene regulation. Cancer Chemother Pharmacol. (2005) 56 Suppl 1:4–9. doi: 10.1007/s00280-005-0102-8 16273365

[10] Kanda N Tamaki K. Estrogen enhances immunoglobulin production by human PBMCs. J Allergy Clin Immunol. (1999) 103:282–8. doi: 10.1016/S0091-6749(99)70503-8 9949320

[11] Taneja V. Sex hormones determine immune response. Front Immunol. (2018) 9:1931. doi: 10.3389/fimmu.2018.01931 30210492 PMC6119719

[12] Scully EP. Sex differences in HIV infection. Curr HIV/AIDS Rep. (2018) 15:136–46. doi: 10.1007/s11904-018-0383-2 PMC5882769 29504062

[13] Birdthistle I Tanton C Tomita A de Graaf K Schaffnit SB Tanser F. Recent levels and trends in HIV incidence rates among adolescent girls and young women in ten high-prevalence African countries: a systematic review and meta-analysis. Lancet Glob Health. (2019) 7:e1521–e40. doi: 10.1016/S2214-109X(19)30410-3 PMC7025003 31607465

[14] Bekker LG Moodie Z Grunenberg N Laher F Tomaras GD Cohen KW. Subtype C ALVAC-HIV and bivalent subtype C gp120/MF59 HIV-1 vaccine in low-risk, HIV-uninfected, South African adults: a phase 1/2 trial. Lancet HIV. (2018) 5:e366–e78. doi: 10.1016/S2352-3018(18)30071-7 PMC6028742 29898870

[15] Tomaras GD Yates NL Liu P Qin L Fouda GG Chavez LL. Initial B-cell responses to transmitted human immunodeficiency virus type 1: virion-binding immunoglobulin M (IgM) and IgG antibodies followed by plasma anti-gp41 antibodies with ineffective control of initial viremia. J Virol. (2008) 82:12449–63. doi: 10.1128/JVI.01708-08 PMC2593361 18842730

[16] Shen X Laher F Moodie Z McMillan AS Spreng RL Gilbert PB. HIV-1 vaccine sequences impact V1V2 antibody responses: A comparison of two poxvirus prime gp120 boost vaccine regimens. Sci Rep. (2020) 10:2093. doi: 10.1038/s41598-020-57491-z 32034163 PMC7005751

[17] Laher F Moodie Z Cohen KW Grunenberg N Bekker LG Allen M. Safety and immune responses after a 12-month booster in healthy HIV-uninfected adults in HVTN 100 in South Africa: A randomized double-blind placebo-controlled trial of ALVAC-HIV (vCP2438) and bivalent subtype C gp120/MF59 vaccines. PloS Med. (2020) 17:e1003038. doi: 10.1371/journal.pmed.1003038 32092060 PMC7039414

[18] Pollara J Hart L Brewer F Pickeral J Packard BZ Hoxie JA. High-throughput quantitative analysis of HIV-1 and SIV-specific ADCC-mediating antibody responses. Cytometry A. (2011) 79:603–12. doi: 10.1002/cyto.a.v79a.8 PMC3692008 21735545

[19] Lin L Finak G Ushey K Seshadri C Hawn TR Frahm N. COMPASS identifies T-cell subsets correlated with clinical outcomes. Nat Biotechnol. (2015) 33:610–6. doi: 10.1038/nbt.3187 PMC4569006 26006008

[20] Haynes BF Gilbert PB McElrath MJ Zolla-Pazner S Tomaras GD Munir Alam S. Immune-correlates analysis of an HIV-1 vaccine efficacy trial. N Engl J Med. (2012) 366:1275–86. doi: 10.1056/NEJMoa1113425 PMC3371689 22475592

[21] Yates NL deCamp AC Korber BT Liao HX Irene C Pinter A. HIV-1 envelope glycoproteins from diverse clades differentiate antibody responses and durability among vaccinees. J Virology. (2018) 92:e01843–17. doi: 10.1128/JVI.01843-17 PMC5874409 29386288

[22] Forthal DN Finzi A. Antibody-dependent cellular cytotoxicity in HIV infection. AIDS. (2018) 32:2439–51. doi: 10.1097/QAD.0000000000002011 PMC6497078 30234611

[23] Barouch DH Alter G Broge T Linde C Ackerman ME Brown EP. Protective efficacy of adenovirus/protein vaccines against SIV challenges in rhesus monkeys. Science. (2015) 349:320–4. doi: 10.1126/science.aab3886 PMC4653134 26138104

[24] Forthal DN Gilbert PB Landucci G Phan T. Recombinant gp120 vaccine-induced antibodies inhibit clinical strains of HIV-1 in the presence of Fc receptor-bearing effector cells and correlate inversely with HIV infection rate. J Immunol. (2007) 178:6596–603. doi: 10.4049/jimmunol.178.10.6596 17475891

[25] Yates NL Liao HX Fong Y deCamp A Vandergrift NA Williams WT. Vaccine-induced Env V1-V2 IgG3 correlates with lower HIV-1 infection risk and declines soon after vaccination. Sci Transl Med. (2014) 6:228ra39. doi: 10.1126/scitranslmed.3007730 PMC4116665 24648342

[26] Atabani S Landucci G Steward MW Whittle H Tilles JG Forthal DN. Sex-associated differences in the antibody-dependent cellular cytotoxicity antibody response to measles vaccines. Clin Diagn Lab Immunol. (2000) 7:111–3. doi: 10.1128/CDLI.7.1.111-113.2000 PMC95833 10618288

[27] Zedan HT Smatti MK Al-Sadeq DW Al Khatib HA Nicolai E Pieri M. SARS-CoV-2 infection triggers more potent antibody-dependent cellular cytotoxicity (ADCC) responses than mRNA-, vector-, and inactivated virus-based COVID-19 vaccines. J Med Virol. (2024) 96:e29527. doi: 10.1002/jmv.29527 38511514

[28] St Clair LA Chaulagain S Klein SL Benn CS Flanagan KL. Sex-differential and non-specific effects of vaccines over the life course. Curr Top Microbiol Immunol. (2023) 441:225–51. doi: 10.1007/978-3-031-35139-6_9 PMC10917449 37695431

[29] Sachdev D Ahmed H Huang Y Gilbert P Rerks-Ngarm S Kaewkugwal J. Sex differences in immune variables in the RV144 trial. AIDS Res Hum Retroviruses. (2014) 30. doi: 10.1089/aid.2014.5410.abstract

[30] Troy JD Hill HR Ewell MG Frey SE. Sex difference in immune response to vaccination: A participant-level meta-analysis of randomized trials of IMVAMUNE smallpox vaccine. Vaccine. (2015) 33:5425–31. doi: 10.1016/j.vaccine.2015.08.032 PMC4581981 26319063

[31] Haralambieva IH Ovsyannikova IG Kennedy RB Larrabee BR Shane Pankratz V Poland GA. Race and sex-based differences in cytokine immune responses to smallpox vaccine in healthy individuals. Hum Immunol. (2013) 74:1263–6. doi: 10.1016/j.humimm.2013.06.031 PMC4170575 23806267

[32] Riggenbach MM Haralambieva IH Ovsyannikova IG Schaid DJ Poland GA Kennedy RB. Mumps virus-specific immune response outcomes and sex-based differences in a cohort of healthy adolescents. Clin Immunol. (2022) 234:108912. doi: 10.1016/j.clim.2021.108912 34968746 PMC8760162

[33] Cook IF. Sexual dimorphism of humoral immunity with human vaccines. Vaccine. (2008) 26:3551–5. doi: 10.1016/j.vaccine.2008.04.054 18524433

[34] Engler RJM Nelson MR Klote MM VanRaden MJ Huang C Cox NJ. Half- vs full-dose trivalent inactivated influenza vaccine (2004-2005): age, dose, and sex effects on immune responses. Arch Intern Med. (2008) 168:2405–14. doi: 10.1001/archinternmed.2008.513 19064822

[35] Aldakak L Huber VM Ruhli F Bender N. Sex difference in the immunogenicity of the quadrivalent Human Papilloma Virus vaccine: Systematic review and meta-analysis. Vaccine. (2021) 39:1680–6. doi: 10.1016/j.vaccine.2021.02.022 33637386

[36] Barnabas RV Brown ER Onono MA Bukusi EA Njoroge B Winer RL. Efficacy of single-dose HPV vaccination among young African women. NEJM Evid. (2022) 1:VIDoa2100056. doi: 10.1056/EVIDoa2100056 PMC9172784 35693874

[37] Xu S Carpenter MC Spreng RL Neidich SD Sarkar S Tenney D. Impact of adjuvants on the biophysical and functional characteristics of HIV vaccine-elicited antibodies in humans. NPJ Vaccines. (2022) 7:90. doi: 10.1038/s41541-022-00514-9 35927399 PMC9352797

[38] Li X Guo S Yang L Hua L Li Z Hao X. Impact of antigens, adjuvants and strains on sexually dimorphic antibody response to vaccines in mice. Biologicals. (2017) 48:47–54. doi: 10.1016/j.biologicals.2017.05.007 28596048

[39] Kayesh MEH Kohara M Tsukiyama-Kohara K. TLR agonists as vaccine adjuvants in the prevention of viral infections: an overview. Front Microbiol. (2023) 14. doi: 10.3389/fmicb.2023.1249718 PMC10764465 38179453

[40] Spiering AE de Vries TJ. Why females do better: the X chromosomal TLR7 gene-dose effect in COVID-19. Front Immunol. (2021) 12:756262. doi: 10.3389/fimmu.2021.756262 34858409 PMC8632002

[41] Berghofer B Frommer T Haley G Fink L Bein G Hackstein H. TLR7 ligands induce higher IFN-alpha production in females. J Immunol. (2006) 177:2088–96. doi: 10.4049/jimmunol.177.4.2088 16887967

[42] Moody MA Santra S Vandergrift NA Sutherland LL Gurley TC Drinker MS. Toll-like receptor 7/8 (TLR7/8) and TLR9 agonists cooperate to enhance HIV-1 envelope antibody responses in rhesus macaques. J Virol. (2014) 88:3329–39. doi: 10.1128/JVI.03309-13 PMC3957956 24390332

[43] Klein SL Pekosz A. Sex-based biology and the rational design of influenza vaccination strategies. J Infect Dis. (2014) 209 Suppl 3:S114–9. doi: 10.1093/infdis/jiu066 PMC4157517 24966191

