# Supplementary figures and images for: Retrospective analysis of sex-disaggregated immune responses to ALVAC-HIV and bivalent subtype C gp120/MF59 HIV vaccines
Source: Front Immunol. 2025 May 14;16:1557009. doi: 10.3389/fimmu.2025.1557009 (PMC12116586; doi:10.3389/fimmu.2025.1557009)

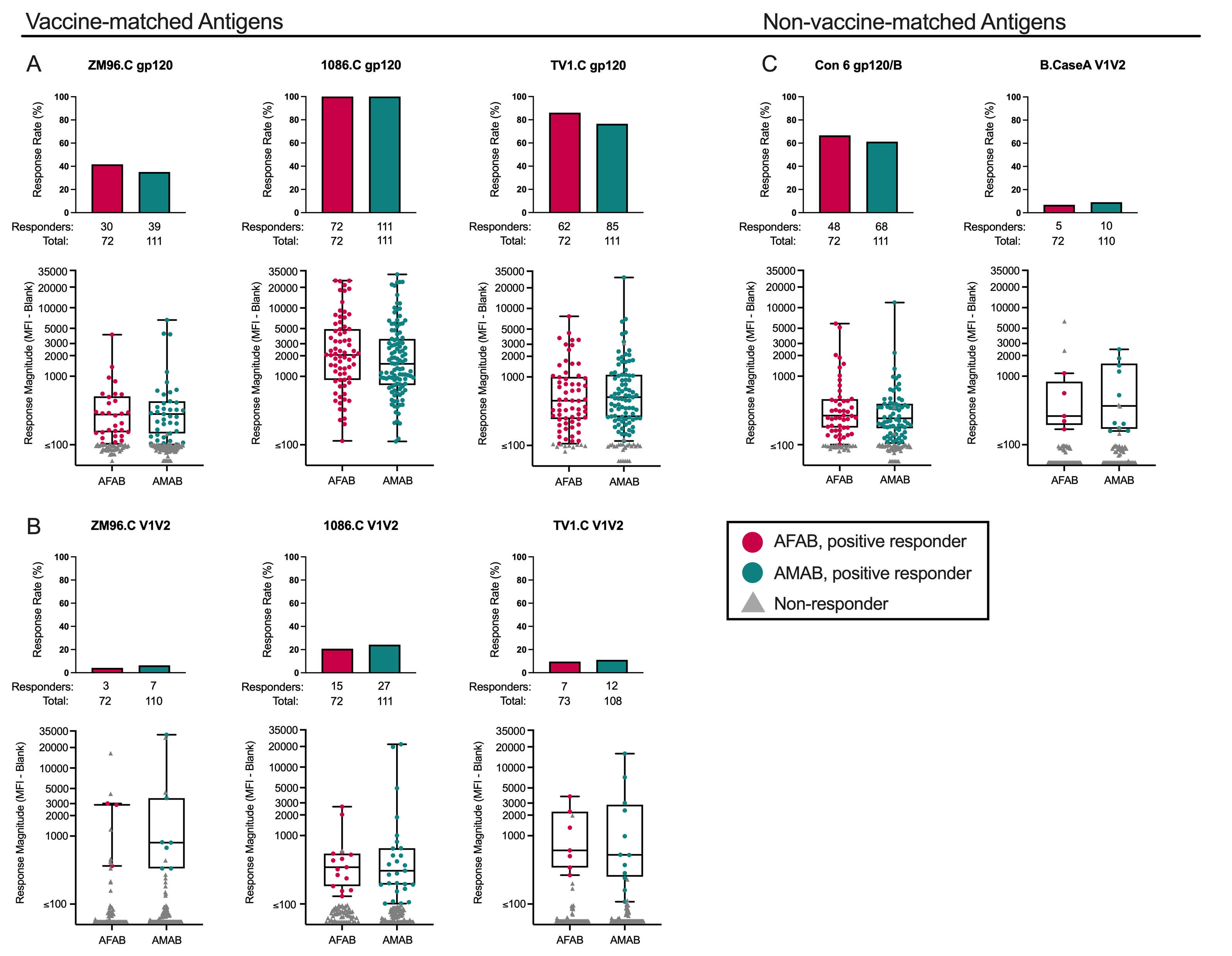

Supplement: Supplementary Figure 1 — IgG3 binding Ab response rates and magnitudes among AFAB and AMAB vaccine recipients. (A–C) There were no sex-based differences in response rates or response magnitudes. Response rates are shown using bar graphs. Boxplots of positive responders show magnitude as MFI-Blank responses. P values compare response rates and magnitudes among positive responders – AFAB positive responders (shown in red circles), AMAB positive responders (shown in dark green circles); negative responders are shown as gray triangles. Adjusted p-values < 0.05 are reported. AFAB, assigned female at birth; AMAB, assigned male at birth; gp120, glycoprotein 120; IgG, immunoglobulin G; V1V2, Variable loops 1 and 2. [file Image1.tiff]
