# Supplementary material for: Retrospective analysis of sex-disaggregated immune responses to ALVAC-HIV and bivalent subtype C gp120/MF59 HIV vaccines
Source: Front Immunol. 2025 May 14;16:1557009. doi: 10.3389/fimmu.2025.1557009 (PMC12116586; doi:10.3389/fimmu.2025.1557009)
Supplement: Supplementary file 2 [file Table1.docx]

**Table S1. IgG and IgG3 binding Antibody, Neutralizing Antibody, ADCC, ADCP, and CD4+ ICS response rates and magnitudes among AFAB and AMAB vaccine recipients**

|  | **Response Rates** | | | | | | **Response Magnitudes** | | | |
| --- | --- | --- | --- | --- | --- | --- | --- | --- | --- | --- |
|  | AFAB | | AMAB | |  |  | AFAB | AMAB |  |  |
|  | n/N | % (95% CI) | n/N | % (95% CI) | *P* | *P*_adj_ | Median (IQR) | Median (IQR) | *P* | *P*_adj_ |
| **Binding IgG Antibodies** | | | | | | | | | | |
| ZM96.C gp120 | 73/73 | 100% (95.0-100) | 112/112 | 100% (96.7-100) | 1 | 1 | 2867.3  (1621.8-4868.5) | 2616.1  (1512.9-4798.9) | 0.44 | 1 |
| 1086.C gp120 | 73/73 | 100% (95.0-100) | 112/112 | 100% (96.7-100) | 1 | 1 | 28489.3  (26115.2-30185.5) | 28289.4  (25828.4-31014.8) | N/A^+^ | N/A^+^ |
| TV1.C gp120 | 73/73 | 100% (95.0-100) | 112/112 | 100% (96.7-100) | 1 | 1 | 12799.5  (6187.5-22097.5) | 12886.6  (5762.1-23069.0) | 0.45 | 1 |
| Con 6 gp120.B | 70/70 | 100% (94.8-100) | 111/111 | 100% (96.7-100) | 1 | 1 | 16294.0  (9923.1-24008.4) | 13792.5  (6680.1-21726.1) | 0.02 | 0.15 |
| ZM96 V1V2 | 31/70 | 44.3% (33.2-55.9) | 57/111 | 51.4% (42.2-60.5) | 0.24 | 1 | 963.5  (544.0-1710.5) | 907.5  (628.5-2874.8) | 0.67 | 1 |
| 1086.C V1V2 | 52/72 | 72.2% (61.0-81.2) | 77/111 | 69.4% (60.3-77.2) | 0.37 | 1 | 1342.5  (598.6-2604.4) | 1090.5  (400.5-2571.5) | 0.19 | 0.93 |
| TV1.C V1V2 | 41/70 | 58.6% (46.9-69.4) | 70/108 | 64.8% (55.4-73.2) | 0.25 | 1 | 1968.5  (590.2-4847.8) | 1526.9  (573.5-3461.0) | 0.35 | 1 |
| B.CaseA V1V2 | 28/72 | 38.9% (28.5-50.4) | 63/111 | 56.8% (47.5-65.6) | 0.01 | 0.08 | 731  (425.6-2047.2) | 484.3  (258.2-1109.8) | 0.04 | 0.26 |
| **Binding IgG3 Antibodies** | | | | | | | | | | |
| ZM96.C gp120 | 30/72 | 41.7% (31.0-53.2) | 39/111 | 35.1% (26.9-44.4) | 0.25 | 1 | 275.9  (152.9-482.4) | 281.0  (152.1-422.2) | 0.59 | 1 |
| 1086.C gp120 | 72/72 | 100% (94.9-100) | 111/111 | 100% (96.7-100) | 1 | 1 | 2056.8  (890.2-4893.6) | 1525.5  (755.2-3532.4) | 0.09 | 0.74 |
| TV1.C gp120 | 62/72 | 86.1% (76.3-92.3) | 85/111 | 76.6% (67.9-83.5) | 0.06 | 0.51 | 449.5  (244.8-982.6) | 506.5  (260.0-1076.0) | 0.77 | 1 |
| Con 6 gp120.B | 48/72 | 66.7% (55.2-76.5) | 68/111 | 61.3% (52.0-69.8) | 0.34 | 1 | 265.6  (177.6-454.5) | 242.6  (177.5-395.4) | 0.22 | 1 |
| ZM96 V1V2 | 3/72 | 4.2% (1.4-11.6) | 7/110 | 6.4% (3.1-12.6) | 0.33 | 1 | 2877.8  (1620.9-2947.4) | 802  (503.1-2212.8) | 0.41 | 1 |
| 1086.C V1V2 | 15/72 | 20.8% (13.1-31.6) | 27/111 | 24.3% (17.3-33.1) | 0.37 | 1 | 344.8  (209.5-534.0) | 306.0  (191.8-650.5) | 0.44 | 1 |
| TV1.C V1V2 | 7/73 | 9.6% (4.7-18.5) | 12/108 | 11.1% (6.5-18.4) | 0.40 | 1 | 610.5  (421.6-1779.8) | 524.3  (272.2-2510.2) | 0.42 | 1 |
| B.CaseA V1V2 | 5/72 | 6.9% (3.0-15.2) | 10/110 | 9.1% (5.0-15.9) | 0.36 | 1 | 262.3  (220.5-564.2) | 367.8  (180.7-1367.3) | 0.57 | 1 |
| **IgG and IgG3 Magnitude-Breadth Scores** | | | | | | | | | | |
| **IgG Antibody Breadth Scores** | | | | | | | | | | |
| V1V2 | 61 | -- | 97 | -- | N/A | N/A | 2.3  (1.5-2.8) | 2.5  (1.6-2.9) | 0.81 | 0.81 |
| gp120 | 73 | -- | 111 | -- | N/A | N/A | 4.2  (4.0-4.3) | 4.1  (3.9-4.2) | 0.22 | 0.53 |
| gp140 | 73 | -- | 110 | -- | N/A | N/A | 4.2  (4.0-4.3) | 4.1  (3.9-4.2) | 0.18 | 0.53 |
| **IgG3 Antibody Breadth Scores** | | | | | | | | | | |
| V1V2 | 72 | -- | 108 | -- | N/A | N/A | 0.44  (0.2-1.1) | 0.39  (0.1-1.1) | 0.35 | 1 |
| gp120 | 72 | -- | 111 | -- | N/A | N/A | 2.5  (2.1-2.7) | 2.4  (2.0-2.8) | 0.46 | 1 |
| gp140 | 72 | -- | 111 | -- | N/A | N/A | 2.6  (2.3-3.0) | 2.5  (2.2-2.9) | 0.36 | 1 |
| **TZM-bl Neutralizing Antibodies** | | | | | | | | | | |
| MW965.26 | 73/73 | 100% (95.0-100) | 110/112 | 98.2% (93.7-99.5) | 0.19 | 0.38 | 333.8  (157.7-690.2) | 229.2  (144.4-479.9) | 0.07 | 0.14 |
| TV1c8.2 | 72/73 | 98.6% (92.6-99.8) | 110/112 | 98.2% (93.7-99.5) | 0.48 | 0.48 | 101.0  (65.0-201.2) | 91.0  (61.2-158.2) | 0.21 | 0.21 |
| **ADCC Responses** | | | | | | | | | | |
| ZM96.C gp120 | 13/73 | 17.8% (10.7-28.1) | 19/112 | 17.0% (11.1-25.0) | 0.48 | 0.48 | 8.8  (5.6-12.6) | 8.2  (5.3-13.1) | 0.26 | 0.26 |
| 1086.C gp120 | 17/73 | 23.3% (15.1-34.2) | 18/112 | 16.1% (10.4-24.0) | 0.19 | 0.38 | 5.8  (1.4-10.5) | 3.5  (0.7-10.9) | 0.05 | 0.10 |
| TV.C gp120 | 42/73 | 57.5% (46.1-68.2) | 33/112 | 29.5% (21.8-38.5) | 8.44e-5 | **0.0003** | 16.1  (9.2-21.9) | 11.2  (5.4-16.2) | 0.009 | **0.03** |
| **ADCP Responses** | | | | | | | | | | |
| 1086 gp140 | 28/28 | 100% (87.9-100) | 43/43 | 100% (91.8-100) | 1 | 1 | 10.5  (9.5-12.3) | 11.9  (10.0-13.3) | 0.91 | 0.91 |
| **IFN-γ and/or IL-2 CD4+ T cells** | | | | | | | | | | |
| ZM96.C gp120 | 25/68 | 36.8% (26.3-48.6) | 60/111 | 54.1% (44.8-63.0) | 0.01 | **0.04** | 0.15  (0.11-0.23) | 0.19  (0.11-0.38) | 0.90 | 1 |
| 1086.C gp120 | 20/68 | 29.4% (19.9-41.1) | 49/111 | 44.1% (35.3-53.4) | 0.03 | 0.05 | 0.12  (0.09-0.16) | 0.15  (0.10-0.26) | 0.91 | 1 |
| TV1.C gp120 | 30/68 | 44.1% (33.0-55.9) | 55/111 | 49.5% (40.4-58.7) | 0.35 | 0.35 | 0.12  (0.09-0.19) | 0.19  (0.12-0.32) | 1 | 1 |
| **Functionality scores for CD4+ T cell responses** | | | | | | | | | | |
| ZM96 gp120 | 36 | -- | 64 | -- | N/A | N/A | 0.16  (0.1-0.2) | 0.15  (0.1-0.2) | 0.40 | 1 |
| 1086.C gp120 | 40 | -- | 64 | -- | N/A | N/A | 0.11  (0.1-0.2) | 0.12  (0.1-0.1) | 0.61 | 1 |
| TV1.C gp120 | 40 | -- | 64 | -- | N/A | N/A | 0.15  (0.1-0.2) | 0.15  (0.1-0.2) | 0.56 | 1 |
| LAI Gag | 40 | -- | 64 | -- | N/A | N/A | 0.009  (0.0-0.0) | 0.008  (0.0-0.0) | 0.23 | 0.92 |
| **Polyfunctionality scores for CD4+ T cell responses** | | | | | | | | | | |
| ZM96 gp120 | 36 | -- | 64 | -- | N/A | N/A | 0.09  (0.1-0.1) | 0.09 (0.1-0.1) | 0.42 | 1 |
| 1086.C gp120 | 40 | -- | 64 | -- | N/A | N/A | 0.06  (0.0-0.1) | 0.06  (0.0-0.1) | 0.50 | 1 |
| TV1.C gp120 | 40 | -- | 64 | -- | N/A | N/A | 0.08  (0.1-0.1) | 0.08  (0.1-0.1) | 0.41 | 1 |
| LAI Gag | 40 | -- | 64 | -- | N/A | N/A | 0.006  (0.0-0.0) | 0.006  (0.0-0.0) | 0.30 | 1 |

Abbreviations: AFAB=Assigned female at birth; AMAB=Assigned male at birth; CI=Confidence interval; gp=glycoprotein; IQR=Interquartile range; V1V2=Variable loops 1 and 2.

+Values exceeded the maximum titer threshold for the assay (>25,000), and thus, statistical comparisons based on sex could not be performed.

**Table S2. CD4+ and CD8+ ICS response rates and magnitudes among AFAB and AMAB vaccine and placebo recipients**

|  | | **Response Rates** | | | | | | **Response Magnitudes** | | | |
| --- | --- | --- | --- | --- | --- | --- | --- | --- | --- | --- | --- |
|  |  | AFAB | | AMAB | |  |  | AFAB | AMAB |  |  |
|  |  | n/N | % (95% CI) | n/N | % (95% CI) | *P* | *P*_adj_ | Median (IQR) | Median (IQR) | *P* | *P*_adj_ |
| **Month 6.5** | **IFN-γ and/or IL-2 CD4+ T cells** | | | | | | | | | | |
|  | CMV pp65 | 63/78 | 80.8% (70.7-88.0) | 104/121 | 86.0% (78.6-91.0) | 0.23 | 0.46 | 0.25  (0.13-0.55) | 0.34  (0.15-0.69) | 0.93 | 1 |
|  | SEB | 74/74 | 100% (95.1-100) | 111/111 | 100% (96.7-100) | 1 | 1 | 13.5  (12.0-16.5) | 14.3  (11.5-17.5) | 0.77 | 1 |
|  | **IFN-γ and/or IL-2 CD8+ T cells** | | | | | | | | | | |
|  | CMV pp65 | 72/83 | 86.7% (77.8-92.4) | 107/122 | 87.7% (80.7-92.4) | 0.46 | 0.93 | 1.1  (0.5-2.4) | 1.0  (0.3-1.8) | 0.15 | 0.31 |
|  | SEB | 79/79 | 100% (95.4-100) | 112/112 | 100% (96.7-100) | 1 | 1 | 12.7  (9.9-15.2) | 12.4  (9.9-15.8) | 0.50 | 0.50 |

Abbreviations: AFAB=Assigned female at birth; AMAB=Assigned male at birth; CI=Confidence interval; gp=glycoprotein; IQR=Interquartile range; V1V2=Variable loops 1 and 2.
